# Phylogenomics of Colombian *Helicobacter pylori* isolates

**DOI:** 10.1186/s13099-017-0201-1

**Published:** 2017-09-11

**Authors:** Andrés Julián Gutiérrez-Escobar, Esperanza Trujillo, Orlando Acevedo, María Mercedes Bravo

**Affiliations:** 1 Grupo de Investigaciones Biomédicas y Genética Humana Aplicada, Programa de Medicina, Universidad de Ciencias Aplicadas y Ambientales, Calle 222 55-37, Bogotá, Colombia; 2 Programa de Doctorado en Ciencias Biológicas, Universidad Javeriana, Carrera 7 40-62, Bogotá, Colombia; 3 Grupo de Investigación en Biología del Cáncer, Instituto Nacional de Cancerología, Calle 1 9-85, Bogotá, Colombia; 4 Grupo de Biofísica y Bioquímica Estructural, Facultad de Ciencias, Pontificia Universidad Javeriana, Carrera 7 40-62, Bogotá, Colombia

**Keywords:** *Helicobacter pylori*, Phylogenomic analysis, Whole genome sequence

## Abstract

**Background:**

During the Spanish colonisation of South America, African slaves and Europeans arrived in the continent with their corresponding load of pathogens, including *Helicobacter pylori*. Colombian strains have been clustered with the hpEurope population and with the hspWestAfrica subpopulation in multilocus sequence typing (MLST) studies. However, ancestry studies have revealed the presence of population components specific to *H. pylori* in Colombia. The aim of this study was to perform a thorough phylogenomic analysis to describe the evolution of the Colombian urban *H. pylori* isolates.

**Results:**

A total of 115 genomes of *H. pylori* were sequenced with Illumina technology from *H. pylori* isolates obtained in Colombia in a region of high risk for gastric cancer. The genomes were assembled, annotated and underwent phylogenomic analysis with 36 reference strains. Additionally, population differentiation analyses were performed for two bacterial genes. The phylogenetic tree revealed clustering of the Colombian strains with hspWestAfrica and hpEurope, along with three clades formed exclusively by Colombian strains, suggesting the presence of independent evolutionary lines for Colombia. Additionally, the nucleotide diversity of *horB* and *vacA* genes from Colombian isolates was lower than in the reference strains and showed a significant genetic differentiation supporting the hypothesis of independent clades with recent evolution.

**Conclusions:**

The presence of specific lineages suggest the existence of an hspColombia subtype that emerged from a small and relatively isolated ancestral population that accompanied crossbreeding of human population in Colombia.

**Electronic supplementary material:**

The online version of this article (doi:10.1186/s13099-017-0201-1) contains supplementary material, which is available to authorized users.

## Background

*Helicobacter pylori* infects 50% of the global population [1, 2] and is the aetiological agent of gastritis, peptic ulcer and stomach cancer [3, 4]. In general, the infection shows intrafamilial spread and is acquired during childhood and established throughout the life of the host [5]. Chronic active gastritis is caused by the bacteria in all infected subjects; between 10 and 15% of cases progress to peptic ulcer, chronic atrophic gastritis, intestinal metaplasia, gastric dysplasia and cancer or to mucosa-associated lymphoid tissue lymphoma [6].

*Helicobacter pylori* has coevolved along with humans since their migration outside of Africa approximately 60,000 years ago [7]. Multi-locus sequence typing (MLST) studies have shown the modern *H. pylori* strains to cluster into different regional populations based on their geographic origin: hpEurope, hpNEAfrica, hpAfrica1, hpAfrica2, hpAsia2, hpSahul and hpEastAsia; hpEastAsia is divided into three subpopulations, hspEAsia, hspMaori and hspAmerind [8–12]. The hspAmerind subpopulation reflects the migration from Asia to the Americas through the Bering Strait that started 12,000 years ago [13]. A much more recent human migration event is the Spanish colonisation of the Americas 500 years ago. During this period, in addition to the Spanish, African slaves also migrated to the American continent. These migrations exposed the native population to new pathogens, including new strains of *H. pylori*, which led to the disappearance of 80% of the native population in the subsequent decades [14, 15].

Multi-locus sequence typing studies of urban *H. pylori* isolates from Colombia have shown that the strains are mainly of the hpEurope type and, in a lower proportion, of the hpAfrica type; the latter type has been found mainly in the Afro-American population living along the coast [16–18]. Similarly to other regions of Latin America, the native strains of the Amerindian populations in Colombia were displaced by European strains in the mestizo population descended from the Amerindians and the Spanish population that arrived on the continent during the Spanish colonisation [16–21].

*Helicobacter pylori* is a bacterium that can display very fast local adaptive processes via mutation and homologous recombination with other strains [22, 23]. These characteristics are evidenced in the study of Shiota et al. [17], who described the existence of a specific population component in strains isolated from the Colombian mestizo population, which suggests that despite their European origin, these strains are clearly differentiated within this population. Recent whole-genome sequence-based studies have shown that in Nicaragua, Mexico and Colombia, *H. pylori* strains have followed unique evolutionary pathways [18]; and that in Colombia, bacterial populations evolve quickly and have formed new subpopulations from a European source [24]. These findings highlight the need for thorough phylogenomic studies to describe the population structure of the Colombian isolates of *H. pylori.*

*Helicobacter pylori* has species-specific genes that are useful as population markers to explore genetic differentiation among strains; among them are *horB* gene, a member of the outer membrane proteins family of *Helicobacter pylori* [25] that encodes a 30-kDa adhesin essential for the tropism of the bacteria towards human gastric epithelium [26], and the *vacA* gene, one of the major virulence factors of the bacterium, that encodes the cytotoxin VacA, which is a factor that induces apoptosis, increases the permeability of gastric cells and suppresses the immune response, among other effects [27].

A total of 103 genomes of *H. pylori* isolates from Colombia were characterised in this study. Phylogenomic and population analyses were performed. The phylogenomic reconstruction allowed the identification of strains associated with both hpEurope and hspWAfrica and, at the same time, the proposal of a new subpopulation, hspColombia, composed of novel genomes.

## Methods

### Bacterial culture and DNA isolation

A total of 115 *cagA*-positive *H. pylori* strains originally isolated between 1998 and 2007 from patients living in Bogotá and Tunja and the surrounding towns were obtained from the *H. pylori* stock collection of the Instituto Nacional de Cancerología in Bogotá, Colombia. A group of 44 of these 115 strains was included in previous genomic studies [18, 24]. Each isolate was obtained from a single colony. The isolates were grown on blood agar plates supplemented with 7% horse serum (Invitrogen, Grand Island, NY), 1% Vitox (Oxoid, Basingstoke, UK), and Campylobacter selective supplement (Oxoid, Basingstoke, UK), at 37 °C for 3 days under microaerophilic conditions. The isolates belonged to patients with different types of gastric pathologies, including benign, mild and severe conditions associated with *H. pylori* infection. Table 1. The histopathological diagnosis was recorded for all voluntary participants. *H. pylori* genomic DNA was obtained from plate cultures of each isolate using a PureLink Genomic DNA Mini Kit (Life Technologies) according to the manufacturer’s instructions.

**Table 1:** Gastric pathologies of patients

Gastric pathology	Number (% of total)
Gastritis (G)	30 (26.1)
Gastritis and duodenal ulcer (G–DU)	5 (4.3)
Atrophic gastritis (AG)	28 (24.3)
Intestinal metaplasia (IM)	30 (26.1)
Intestinal metaplasia and duodenal ulcer IM–DU	2 (1.7)
Gastric adenocarcinoma (GA)	20 (17.4)
Total	115 (100.0)

### Library preparation and genome sequencing

Libraries were prepared using a Nextera XT DNA Sample Preparation Kit (Illumina, San Diego, CA, USA) with 1 ng of DNA according to the manufacturer’s protocol (Nextera XT protocol, Version October 2012) and sequenced using a MiSeq Personal Sequencer (Illumina, San Diego, CA, USA). Sequencing reactions were performed using MiSeq v2. Chemistry (Illumina, San Diego, CA, USA).

### Genome assembly and annotation

The readings were processed to remove adapters and low-quality regions. Then, reading errors were corrected using the SGA algorithm [28]. The contigs were assembled using the IDBA-UD algorithm [29], and the scaffolds were assembled using the SSPACE tool [30]. These programs were used according to the instructions of the A5-miseq pipeline [31], which was developed specifically for the Illumina platform and for small genomes of haploid organisms. Finally, the genomes were annotated in RAST [32].

### Multi-locus sequence typing (MLST) analysis

The 103 genomes of Colombian isolates were annotated using http://pubmlst.org/helicobacter. Concatenated nucleotide sequences of seven housekeeping genes *atpA, efp, mutY, ppa, trpC, ureI,* and *yphC* from 103 Colombian isolates, and 163 reference were downloaded from the PubMLST database. The reference sequences were: hpEurope: 82 sequences; hpAfrica1: 16 sequences; hpWestAfrica: 23 sequences; hspSouthIndia: 2 sequences; hpEastAsia: 8 sequences; and hspAmerind: 7 sequences. The concatenated nucleotide sequences were aligned using Muscle software [33]. Phylogenetic analyses were conducted in Mega V7 using T92+G+I (Tamura model with Gamma function and Invariable sites) [34]. Bootstrap analysis was performed with 1000 replications, and Phylogenetic tree was edited with iTol v3 [35].

### Phylogenomic reconstructions

The genomes sequenced in this study were aligned with 34 *H. pylori* reference strains from the National Center for Biotechnology Information (NCBI) databases using the Gegenees v2.21 tool. This tool uses an algorithm to align genomic fragments and compares them by Blastn [36, 37]. The fragment size was 200 bp, and the sliding step size was 100 bp. The average sequence similarity was set at 40% to generate a genomic similarity matrix, which was then exported in.nex format. This file was analysed using Splitstree4 v4.14.5 software [38], which generated a rootless tree using the NJ algorithm. Then, the file was edited using iTool v3 software [35]. The core genome SNP analysis was performed using the KSNP v3.0 program [39], this suit has been used for SNP identification and phylogenetic analysis of *H. pylori* strains [40]. K-chooser tool was used to determine the k-mer value. It was 21 and 141 k-mers were indentified in the core. The final phylogenetic tree was a consensus between 100 parsimonious trees. Lastly, a gen cluster analysis was performed using the GET_HOMOLOGUES v3.06 [41] to extract the *horB* and *vacA* genes for population analysis.

### Virulence genes

*HorB* and *vacA* virulence genes were used as population markers because they are present in most isolates, play key roles in the physiopathology of the infection and are species-specific. A total of 137 nucleotide sequences were obtained for each gene, and the sequences were aligned using the Muscle program [33]. The files containing the alignments were analysed using MEGA 6.1 software [34], with which the evolutionary models were determined, and the phylogenetic reconstructions with 1000 bootstrap repetitions were performed using the NJ algorithm [42]. In addition, Tajima’s D test [43] was performed to detect the effects of natural selection on the sequences. Due to the extreme diversity of the *vacA* gene, the Gblocks tool [44] was used to edit the alignment of this gene and to choose its most parsimonious regions.

The marker genes were divided into populations according to the pathology associated with the strain from which they were sequenced. To determine the genetic diversity of the markers, the following population statistics were obtained: number of haplotypes (H), haplotype diversity (Hd), nucleotide diversity (Pi) and average number of nucleotide differences (k). Genetic heterogeneity and genetic flow were evaluated using the Snn, GammaST and Fst tests in the DnaSP 5.10 software [45]. Finally, heterogeneity tests were applied to population pairs.

## Results

A total of 115 *H. pylori* isolates were included in the study; twelve genomes showing low sequence quality were excluded. The 103 remaining genomes showed a mean of 85 contigs, 127× coverage, 1.65 MB in size, 39% G+C and 1647 genes (Additional file 1: Table S1).

**Fig. 1:**
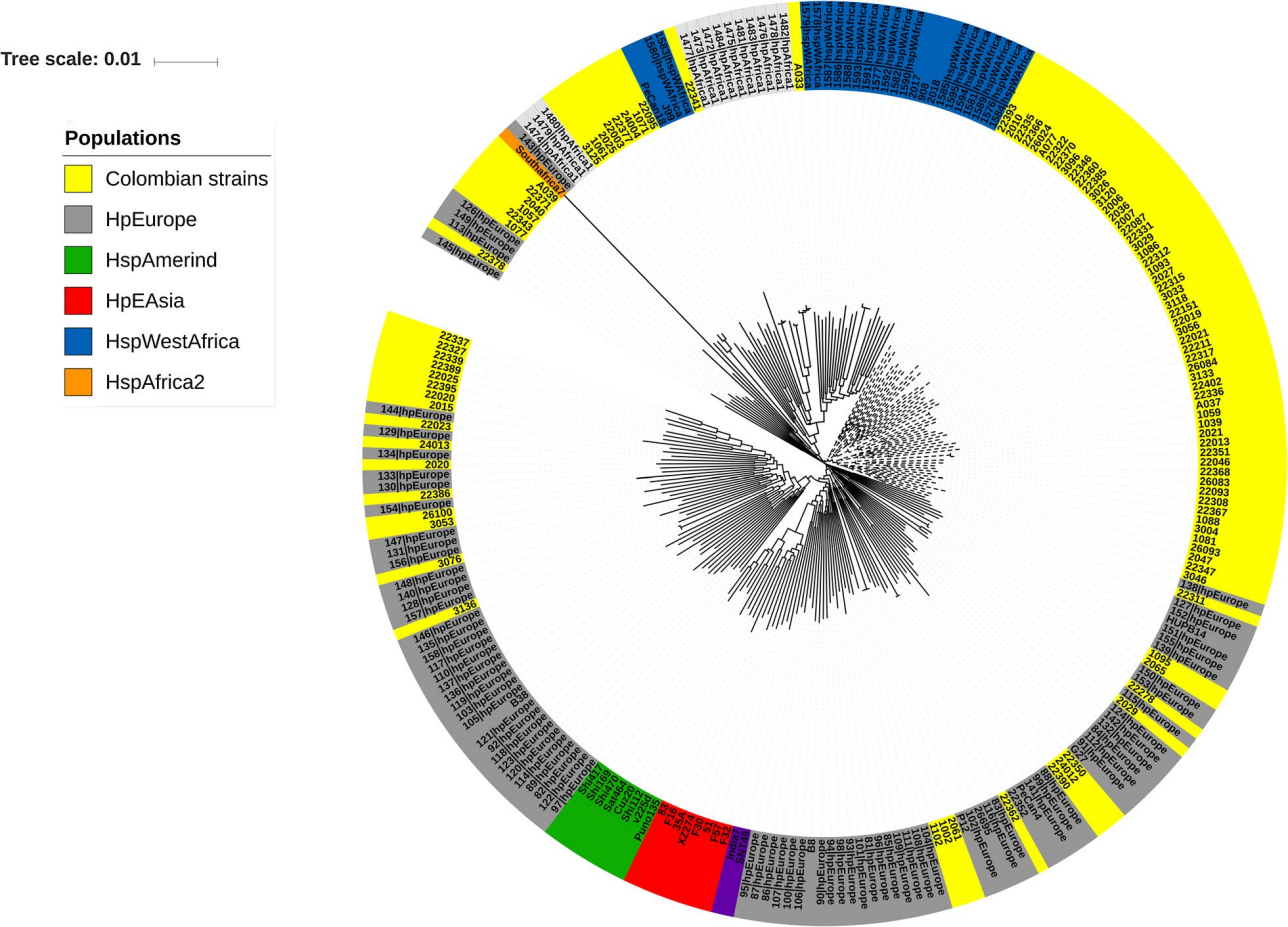
Phylogenetic analyses of 103 Colombian strains and 163 worldwide reference *H. pylori s*equences using MLST

The phylogenetic tree based on MLST sequences of the 103 Colombian strains and 163 reference strains is showed in Fig. 1. Among the 103 Colombian strains eight claded with hpAfrica1; 55 strains were scattered among hpEurope clades. Interestingly the remaining 40 Colombian strains formed three independent clades, suggesting that in Colombia the bacterium has evolved in at least two independent lines with evidence of multiple duplications possibly due to recombination. None of the Colombian strains clustered with the hspAmerind or hpAsia populations, suggesting that the hspAmerind subpopulation has been lost in urban Colombian populations.

**Fig. 2:**
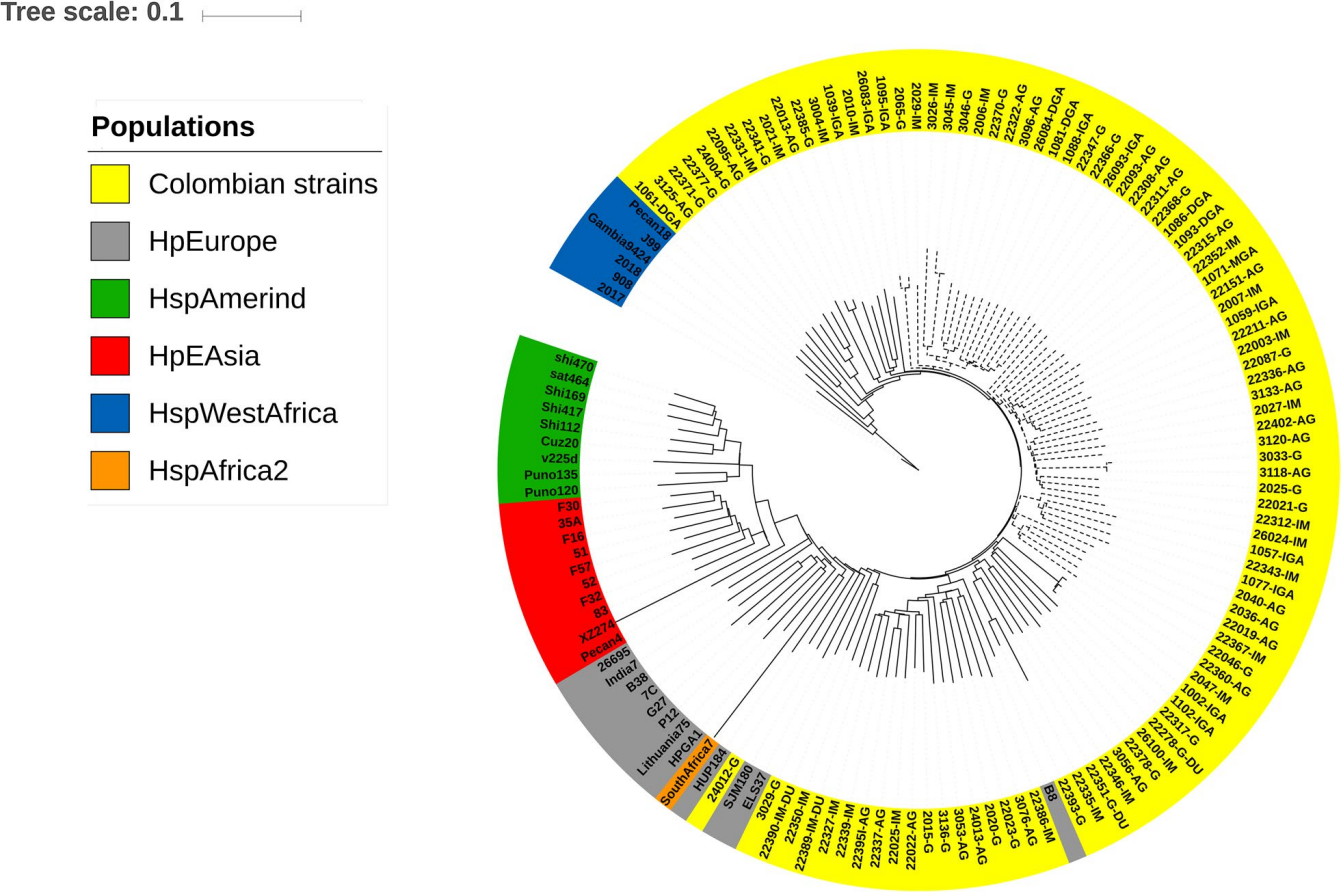
Phylogenomics analyses of *H. pylori* isolates from Colombia analyzed with Gegenees v2.2.1 software. Reference genomes: hpEurope: 26695, B8, B38, ELSE37, G27, HPAG1, Lithuania75, P12 and SJM180; hpWestAfrica: 908, 2017, 2018, Gambia94-24, J99 and PeCan18; hpAfrica1: Southafrica 7 and Southafrica 20; hspSouthIndia genomes: India7 and SNT49; hpEastAsia: 35A, 51, 83, F16, F30, F32, F57 and XZ274; and hspAmerind: Cuz20, PeCan4, Puno135, Sat464, Shi112, Shi169, Shi417, Shi470 and v225d. Dashed lines represent the independent evolutionary lines of Colombian strains

**Fig. 3:**
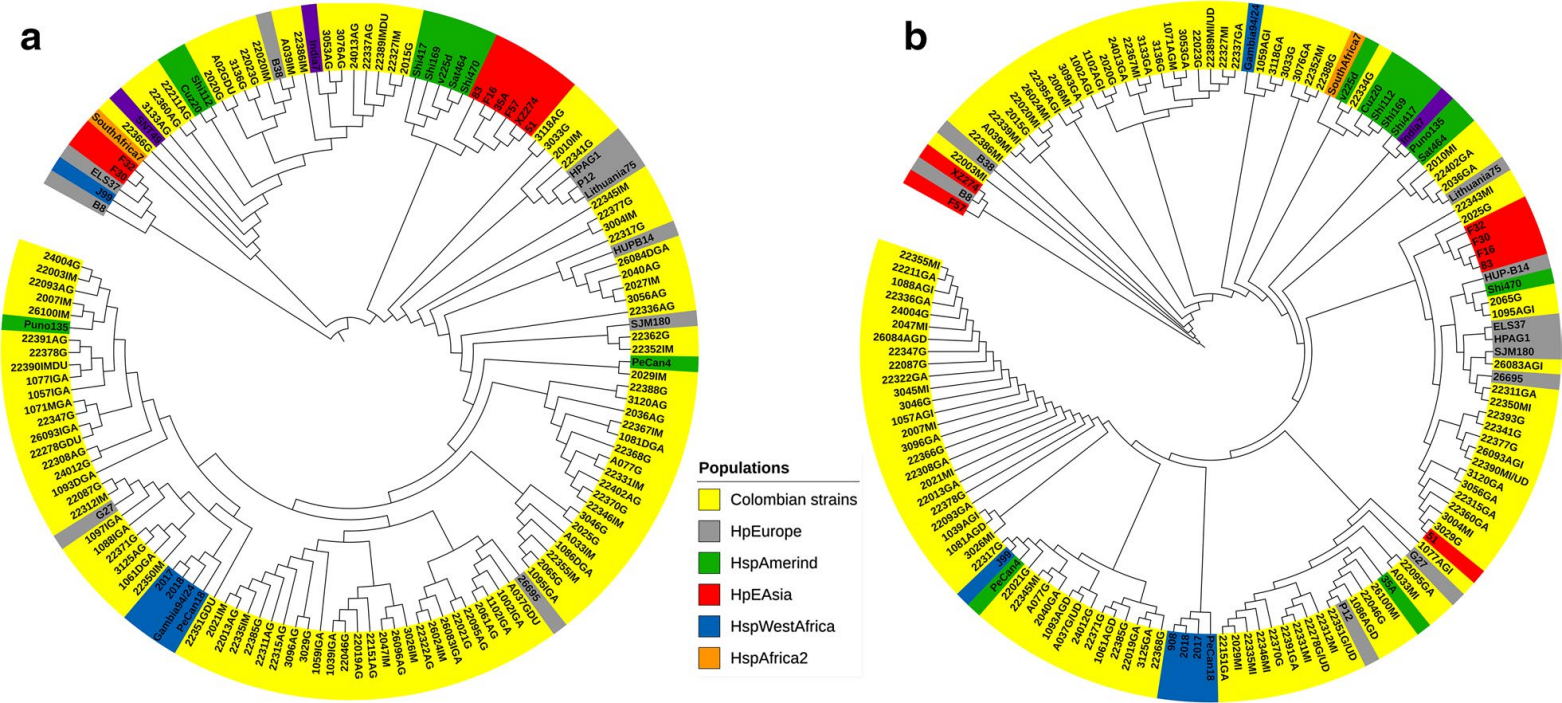
Phylogenetic analysis of virulence factors. **a** VacA, the phylogeny was inferred from 138 sequences using maximum likelihood based on the GTR+G model. Only the first positions were included, and all gaps were removed. **b** HorB, the phylogeny was inferred from 140 sequences using the NJ algorithm, and the distance was computed using the Kimura 2-parameter method with a gamma distribution of 1; all gaps were removed. A total of 1000 bootstrap repetitions were used for all the reconstructions as statistical support

**Fig. 4:**
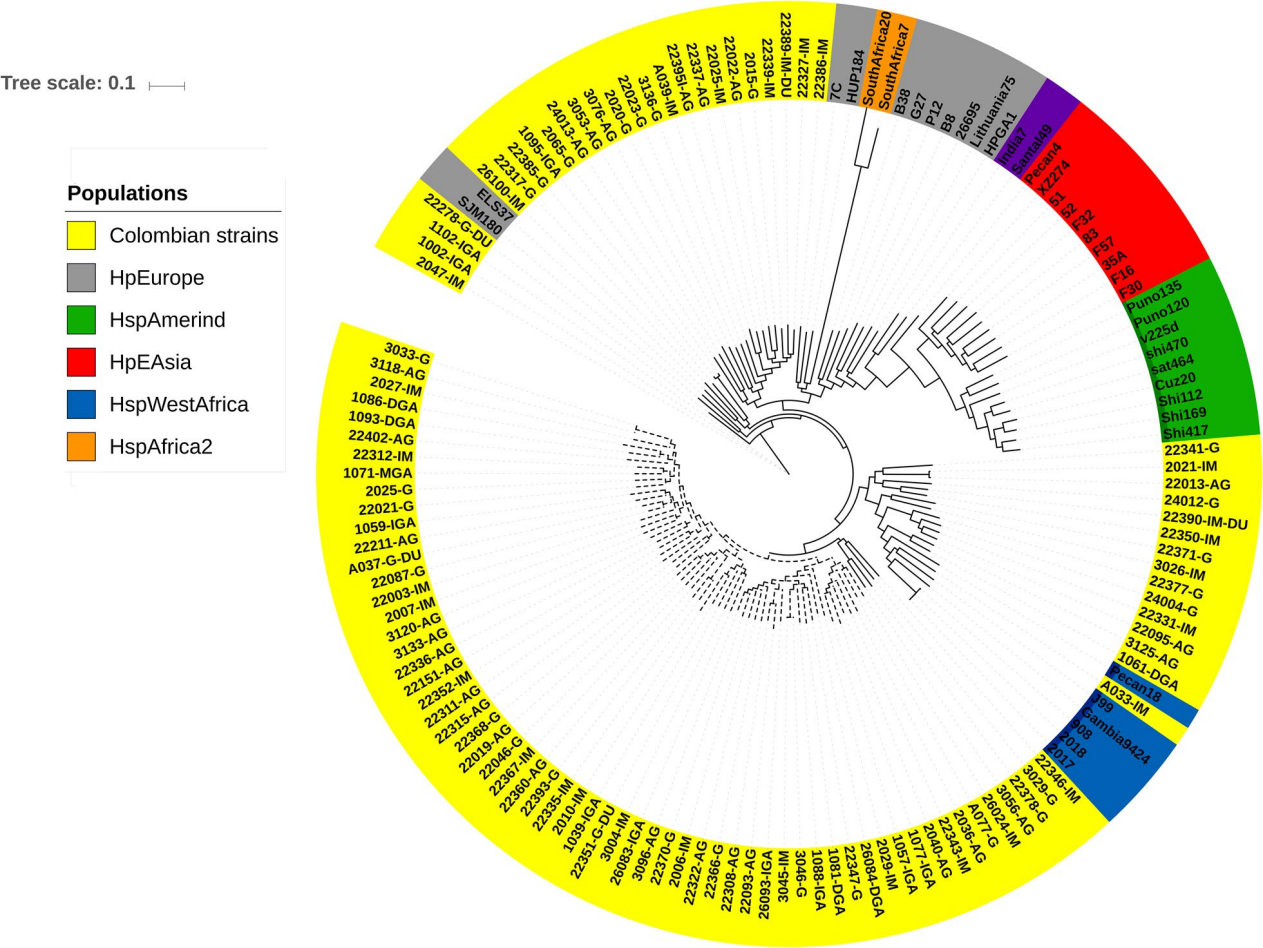
Core genome SNP tree of *H. pylori* isolates from Colombia analyzed with kSNP v3.0 software suite. Dashed lines represent the independent evolutionary lines of Colombian strains

Phylogenomic analysis of the 34 *H. pylori* reference strains revealed a phylogeographic structure similar to that established by MLST studies (Additional file 2: Figure S1) [8]. When the 103 Colombian genomes were added, in concordance with MLST, none clustered with the hspAmerind or hpAsia populations. Fourteen Colombian genomes formed a well-differentiated clade with the genomes of the HspWestAfrica subpopulation, and thirty claded with genomes of HpEurope population. Around 50% of the genomes of Colombian isolates were clustered exclusively in three clades, suggesting the existence of independent evolutionary lines for Colombia (Fig. 2). In agreement with this result, the phylogenetic trees for *vacA* and *horB* genes also showed clades formed exclusively by Colombian isolates (Fig. 3). A core genome phylogenetic tree was constructed to corroborate the independence of Colombian lineages (Fig. 4), it showed Colombian strains grouped in differentiated clades evidencing the presence of specific polymorphisms.

Nucleotide diversity, number of haplotypes, and average number of nucleotide differences were lower for *vacA* and *horB* genes from Colombian strains in comparison with the reference pool; recombination was lower only for *vacA*, while haplotype diversity was equally high for all populations. These results suggest that Colombian isolates descended from a small and isolated population (Table 2).

**Table 2:** Analysis of genetic diversity, differentiation and genetic flow in total populations

Gen	Population	GDIV						GDIF	GFL	
		D	H	Hd	Pi	k	RM	Snn	GammaST	FsT
*vacA*	Col. and Ref.	−0.40104	126	0.999	0.106	64.83	92	0.283*	0.165 (2.52)	0.074 (6.19)
	Col.		99	0.999	0.061	40.77	98			
	Ref.		33	0.997	0.171	120.28	112			
*horB*	Col. and Ref.	−1.99605	120	0.996	0.051	37.21	48	0.339*	0.161 (2.59)	0.105 (4.25)
	Col.		91	0.995	0.031	27.75	43			
	Ref.		33	0.995	0.104	76.4	34			

The Nm number for each genetic flow test is shown in parentheses. A total of 138 sequences were used for *vacA* and 140 for *horB*. Analyses were performed in DnaSP v 5.10

*GDIV* genetic diversity, *GDIF* genetic differentiation, *GFL* genetic flows, *Col* Colombian strains, *Ref* reference strains, *D* Tajima’s test, *P* compared populations, *H* number of haplotypes, *Hd* haplotype diversity, *Pi* nucleotide diversity, *k* genetic load

* *P* = 0.001

**Fig. 5:**
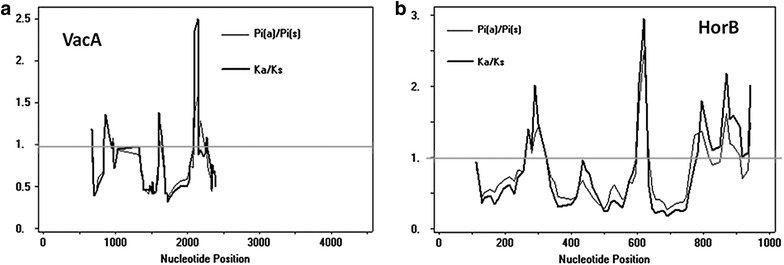
Analysis of Ka/Ks versus nucleotide position. **a** VacA, the analysis was inferred from 138 sequences and all gaps were removed. **b** HorB, the analysis was inferred from 140 sequences and all the gaps were removed. The analyses were performed using DnaSP v 5.10

The Snn test indicated that the Colombian populations, according to *vacA* and *horB,* are well differentiated from the reference pool, and the Nm value in the genetic flow tests indicates that this flow is constant in the population (Table 1). Both genes showed areas with Ka/Ks values above 1 with similar patterns, which suggests that these areas are under selective pressure (Fig. 5), Tajima test was negative but not significant. The pairwise FsT comparison for each gene showed significant population isolation between the Colombian strains and the hspAmerind and hpAsia subpopulations. No genetic differentiation was observed between HpEurope and hspWestAfrica and Colombian strains in *horB* and *vacA* genes. However, *vacA* gene from the Colombian strains isolated from subjects with intestinal or diffuse gastric cancer was differentiated from hpEurope (Table 3). These results together indicate that the Colombian isolates of *Helicobacter pylori* have evolved independently under purifying selection.

**Table 3:** Pairwise analysis of genetic differentiation

Populations	FsT		GammaST	
	*horB*	*vacA*	*horB*	*vacA*
Amerind				
G vs. hspAmerind	*0.297*	*0.204*	*0.131*	*0.131*
GA vs. hspAmerind	*0.313*	*0.186*	*0.151*	*0.116*
IM vs. hspAmerind	*0.28*	*0.214*	*0.132*	*0.141*
IGA vs. hspAmerind	*0.316*	*0.334*	*0.232*	*0.262*
DGA vs. hspAmerind	*0.33*	*0.367*	*0.285*	*0.240*
G/DU vs. hspAmerind	*0.337*	*0.112*	*0.280*	*0.133*
IM/DU vs. hspAmerind	*0.303*	−0.062	*0.269*	*0.110*
Europe				
G vs. HpEurope	0.049	0.062	0.079	0.071
GA vs. HpEurope	0.056	0.063	0.084	0.069
IM vs. HpEurope	0.037	0.064	0.073	0.073
IGA vs. HpEurope	0.059	*0.119*	0.090	*0.109*
DGA vs. HpEurope	0.051	*0.143*	0.074	0.098
G/DU vs. HpEurope	0.044	0.014	0.057	0.052
IM/DU vs. HpEurope	0.082	−0.008	0.050	0.052
Asia				
G vs. HpAsia	*0.163*	*0.27*	*0.167*	*0.200*
GA vs. HpAsia	*0.172*	*0.266*	*0.174*	*0.190*
IM vs. HpAsia	*0.163*	*0.274*	*0.167*	*0.210*
IGA vs. HpAsia	*0.158*	*0.326*	*0.163*	*0.275*
DGA vs. HpAsia	*0.162*	*0.355*	*0.133*	*0.239*
G/DU vs. HpAsia	*0.15*	*0.214*	0.099	*0.170*
IM/DU vs. HpAsia	*0.153*	*0.121*	0.083	*0.131*
Africa				
G vs. HpAfrica	0.055	0.016	0.054	0.083
GA vs. HpAfrica	0.076	0.009	0.063	0.075
IM vs. HpAfrica	0.077	0.007	0.063	0.083
IGA vs. HpAfrica	0.095	0.059	*0.132*	*0.148*
DGA vs. HpAfrica	0.013	0.051	*0.133*	*0.134*
G/DU vs. HpAfrica	0.077	−0.058	*0.197*	*0.109*
IM/DU vs. HpAfrica	0.082	−0.093	*0.229*	*0.148*

A total of 138 sequences were used for vacA and 140 for horB. Analyses were performed in DnaSP v 5.10. Significant values are highlighted in italics

*G* gastritis, *GA* gastric adenocarcinoma, *IM* intestinal metaplasia, *IGA* intestinal gastric adenocarcinoma, *DGA* diffuse gastric adenocarcinoma, *G/DU* gastritis + duodenal ulcer, *IM/DU* metaplasia + duodenal ulcer

## Discussion

The phylogenetic analysis based on the complete *H. pylori* genome showed that approximately half of the strains isolated from the Colombian mestizo population clustered with the hpEurope and hspWestAfrica clades. This finding was also reported in previous MLST studies conducted in this population [16–18], and represents the introduction of European strains by the Spanish during the conquest of America and of strains from the West of Africa due to the subsequent arrival of African slaves. The remaining strains analysed constituted three independent clades consisting exclusively of Colombian strains, suggesting the presence of independent evolutionary lines in the country. Mestizos have their own genetic components, which are relatively new in human history [46]. The admixture between mestizos allowed the recombination of hpEurope type strains and their adaptation to a new host, generating the hspColombia subpopulation. This type of adaptive event has also been reported in Senegal [47].

The phylogenomic reconstruction revealed that the Colombian isolates are not related to hspAmerind type genomes; this finding is consistent with previous studies showing that no Asian components are found in the population structure of *H. pylori* [16–18]. Prior to colonisation, the region was dominated by hspAmerind strains, which arrived on the American continent through the Bering Strait [48]. This subpopulation has been reported in Amerindians in Peru [13], Mexico, Venezuela and Colombia [49]; however, currently in urban mestizo populations the hspAmerind strains have been replaced by hpEurope strains [16–18, 49–52]. The new niche that emerged from the conquest was a mixture of haplotypes (European, Amerindian and African) that allowed competition among circulating strains and put the hspAmerind subpopulation at a disadvantage, ultimately causing it to disappear from the mestizo population.

The emergence of independent evolutionary lines for *H. pylori* in Colombia over a relatively short time, from the Spanish conquest in 1492 to the present, can be explained by the adaptive capacity of *H. pylori*. The population structure of the bacterium is panmictic and naturally competent, that is, the bacterium can take genetic material from the external environment, incorporate it into its genome and express it [53]. In addition, *H. pylori* shows great genetic diversity: the gene content and order vary among strains, its genes have mosaic structures, and the most conserved genes are highly variable at the DNA sequence level [53]. These features allow the bacteria to undergo rapid microevolutionary changes [22, 23, 54] generating new population subtypes such as those reported in Malaysia [55] and Arabia [56] and now in Colombia, where the hspColombia subtype is proposed.

The hspColombia subtype is also supported by the results of the phylogenetic and population analyses performed on *horB* and *vacA* virulence genes. The phylogenetic reconstruction of each gene indicated the presence of specific Colombian clades, suggesting the existence of independent evolutionary processes for the Colombian isolates. When the full populations were assessed for each marker, several features pointing to this conclusion were found: (1) low nucleotide diversity with high haplotype diversity was found; (2) high genetic differentiation with constant genetic flow was determined; (3) both genes showed areas under strong selective pressure, likely because these genes are immunogenic; and (4) the Colombian populations are significantly less diverse than the reference genomes. It is possible that Colombian strains may have evolved to be more virulent than the European ones, considering that *vacA* is an important virulence factor and that Colombian gastric cancer-associated strains differ from hpEurope in their *vacA* sequence. Further studies are warranted to test this possibility.

## Conclusion

HspColombia is characterised by being genetically differentiated from the hspAmerind and hpAsia populations, showing an African component that has been assimilated during the evolutionary process and having as a common ancestor the hpEurope type.

## Additional files

**Additional file 1.** Genome statistics of the sequenced Colombian *H. pylori* isolates. Table showing the genome statistics of the sequenced Colombian *H. pylori* isolates.

**Additional file 2.** Phylogenomics reconstruction using 31 reference strains. Phylogenomic reconstruction using the following populations: hpEurope genomes 26695, B8, B38, ELSE37, G27, HPAG1, Lithuania75, P12 and SJM180; hpWestAfrica genomes 908, 2017, 2018, Gambia94-24, J99 and PeCan18; hpAfrica2 (grey) genomes Southafrica 7 and Southafrica 20; hspSouthIndia genomes India7 and SNT49; hpEastAsia genomes 35A, 51, 83, F16, F30, F32, F57 and XZ274; and hspAmerind genomes Cuz20, PeCan4, Puno135, Sat464, Shi112, Shi169, Shi417, Shi470 and v225d.
