# Supplementary figures and images for: Phylogenomics of Colombian Helicobacter pylori isolates
Source: Gut Pathog. 2017 Sep 11;9:52. doi: 10.1186/s13099-017-0201-1 (PMC5594506; doi:10.1186/s13099-017-0201-1)

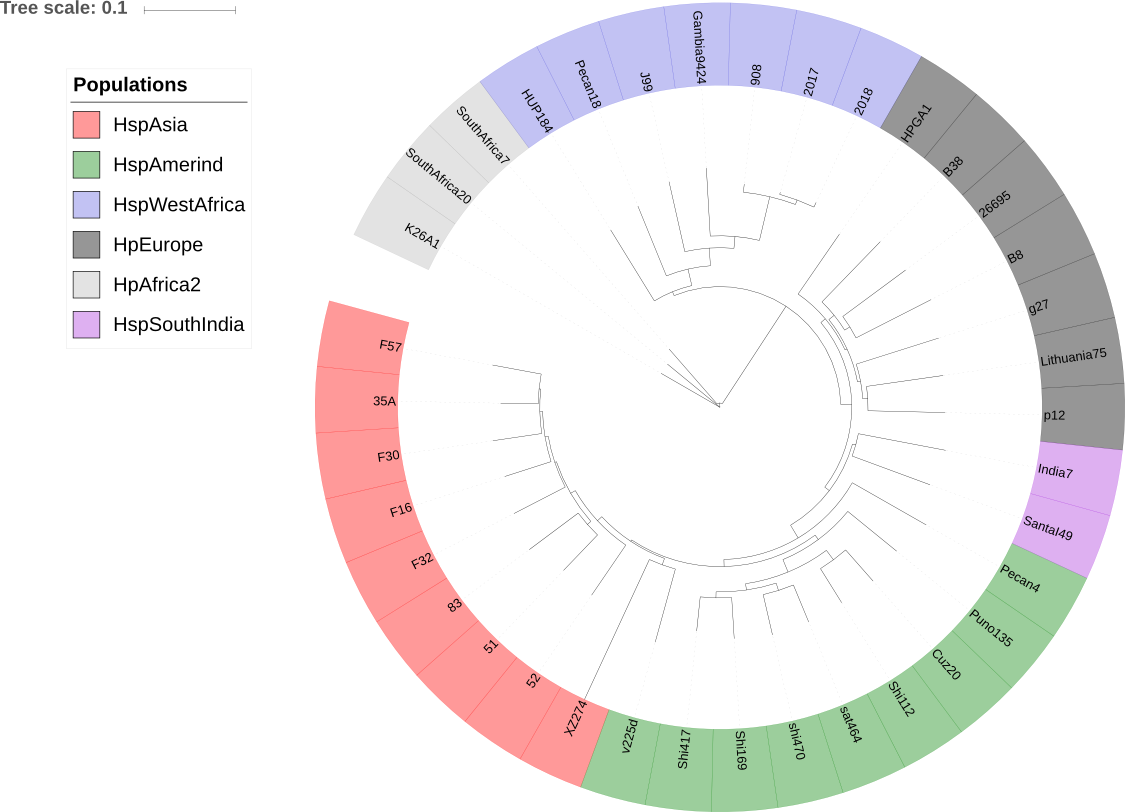

Supplement: Supplementary file 2 — Additional file 2. Phylogenomics reconstruction using 31 reference strains. Phylogenomic reconstruction using the following populations: hpEurope genomes 26695, B8, B38, ELSE37, G27, HPAG1, Lithuania75, P12 and SJM180; hpWestAfrica genomes 908, 2017, 2018, Gambia94-24, J99 and PeCan18; hpAfrica2 (grey) genomes Southafrica 7 and Southafrica 20; hspSouthIndia genomes India7 and SNT49; hpEastAsia genomes 35A, 51, 83, F16, F30, F32, F57 and XZ274; and hspAmerind genomes Cuz20, PeCan4, Puno135, Sat464, Shi112, Shi169, Shi417, Shi470 and v225d. [file 13099_2017_201_MOESM2_ESM.tiff]
